# Corrigendum: The Tumor Suppressor Role of Zinc Finger Protein 671 (*ZNF671*) in Multiple Tumors Based on Cancer Single-Cell Sequencing

**DOI:** 10.3389/fonc.2020.00339

**Published:** 2020-03-10

**Authors:** Jian Zhang, Jianli Luo, Huali Jiang, Tao Xie, Jieling Zheng, Yunhong Tian, Rong Li, Baiyao Wang, Jie Lin, Anan Xu, Xiaoting Huang, Yawei Yuan

**Affiliations:** ^1^Department of Radiation Oncology, Affiliated Cancer Hospital & Institute of Guangzhou Medical University, Guangzhou, China; ^2^State Key Laboratory of Respiratory Diseases, Guangzhou Institute of Respiratory Disease, Affiliated Cancer Hospital & Institute of Guangzhou Medical University, Guangzhou, China; ^3^Department of General Disease, Health Center of Shuichun Town, Shanwei, China; ^4^Department of Cardiovascularology, Tungwah Hospital of Sun Yat-sen University, Dongguan, China; ^5^Department of Radiation Oncology, Nanfang Hospital, Southern Medical University, Guangzhou, China

**Keywords:** *ZNF671*, tumor suppressor, solid tumor, single-cell sequencing, data mining

In the published article, there was an error in affiliation 4. Instead of “Department of Cardiovascularology, the Affiliated Donghua Hospital of Sun Yat-sen University, Guangzhou, China”, it should be “Department of Cardiovascularology, Tungwah Hospital of Sun Yat-sen University, Dongguan, China.”

The authors apologize for this error and state that this does not change the scientific conclusions of the article in any way. The original article has been updated.

